# Exploring the causal correlations between 486 serum metabolites and systemic lupus erythematosus: a bidirectional Mendelian randomization study

**DOI:** 10.3389/fmolb.2023.1281987

**Published:** 2023-11-09

**Authors:** Li Li, Wenyu Li, Qing Ma, Youkun Lin, Zhezhe Cui

**Affiliations:** ^1^ Department of Dermatology and Venereology, The First Affiliated Hospital of Guangxi Medical University, Nanning, China; ^2^ Guangxi Key Laboratory of Major Infectious Disease Prevention and Control and Biosafety Emergency Response, Guangxi Centre for Disease Control and Prevention, Nanning, China

**Keywords:** metabolites, systemic lupus erythematosus, correlations, Mendelian randomization, bidirectional

## Abstract

**Objective:** The observational association between circulating metabolites and systemic lupus erythematosus (SLE) has been well documented. However, whether the association is causal remains unclear. In this study, bidirectional Mendelian randomization (MR) was introduced to analyse the causal relationships and possible mechanisms.

**Methods:** We conducted a two-sample bidirectional MR study. A genome-wide association study (GWAS) with 7,824 participants provided data on 486 human blood metabolites. Outcome information was obtained from a large-scale GWAS summary, which contained 5,201 single nucleotide polymorphisms (SNPs) cases and 9,066 control cases of Europeans and yielded a total of 7,071,163 SNPs. The inverse variance weighted (IVW) model was recruited as the primary two-sample MR analysis approach, followed by sensitivity analyses such as the heterogeneity test, horizontal pleiotropy test, leave-one-out analysis, and linkage disequilibrium score (LDSC) regression.

**Results:** In this study, we discovered that 24 metabolites belonging to the lipid, carbohydrate, xenobiotic and amino acid superpathways may increase the risk of SLE occurrence (*p* < 0.05). In addition, the metabolic disorders of 51 metabolites belonging to the amino acid, energy, xenobiotics, peptide and lipid superpathways were affected by SLE (*p* < 0.05). Palmitoleate belonging to the lipid superpathway and isobutyrylcarnitine and phenol sulfate belonging to the amino acid superpathway were factors with two-way causation. The metabolic enrichment pathway of bile acid biosynthesis was significant in the forward MR analysis (*p* = 0.0435). Linolenic acid and linoleic acid metabolism (*p* = 0.0260), betaine metabolism (*p* = 0.0314), and glycerolipid metabolism (*p* = 0.0435) were the significant metabolically enriched pathways in the reverse MR analysis.

**Conclusion:** The levels of some specific metabolites may either contribute to the immune response inducing SLE, or they may be intermediates in the development and progression of SLE. These metabolites can be used as auxiliary diagnostic tools for SLE and for the evaluation of disease progression and therapeutic effects.

## 1 Introduction

The prevalence of systemic lupus erythematosus (SLE), a complex autoimmune multisystemic disease of great clinical heterogeneity, exhibits a wide range with rates varying between 29 and 7,713 per 100,000 individuals (Barber et al., 2021). The heterogeneity of SLE is not only different in patients with different individuals, different genders, different ages and different course of disease, but also in organ damage and corresponding manifestations, slow onset, overlapping damage, response to treatment, outcome and prognosis, even in identical twins (Bhaskar and Nagaraju, 2019; Wu et al., 2020). As autoimmunity starts before its clinical manifestations, the long time period between the onset of autoimmunity and final diagnosis means that some patients with SLE may develop irreversible, severe organ damage and even death (Nashi and Shmerling, 2021). Early diagnosis and treatment have become an urgent issue in SLE management (Ugarte-Gil et al., 2019). Because of the clinical complexity of lupus, identifying sensitive and specific biomarkers for its diagnosis and monitoring has been challenging. Therefore, the discovery of specific biomarkers is of great significance for the prognosis of SLE.

In the past decade, an increasing number of studies have found an association between metabolomics and SLE and other immune diseases, which provides new ideas for metabolic-derived biomarkers. Metabolomics is the study of small-molecule metabolites in samples or organs of living organisms. Cellular metabolic programs could affect the immune response by regulating the activation, proliferation, and differentiation of innate and adaptive immune cells. Many studies have shown that the dysregulation of the immune system is associated with changes in metabolite profiles (Teng et al., 2020). Metabolites can regulate the process of immune diseases by targeting the corresponding receptors (Wu et al., 2023a).

Metabolism is known for its intricate complexity at the biochemical level. Recent studies have added levels of complexity showing “moonlighting” functions of some metabolic enzymes, such as glyceraldehyde-3-phosphate dehydrogenase (GAPDH) regulating Interferon γ (IFN-γ) production, and metabolites, such as succinate or itaconate, with previously unsuspected immune signalling functions (Chang et al., 2013; Murphy and O'Neill, 2018). Therefore, screening out certain unreported metabolites associated with SLE by Mendelian randomization (MR) analysis has enlightening implications for exploring the mechanisms of pathophysiology.

While metabolic abnormalities affect the development of SLE, the disease also increases the metabolic burden. In an observational study (Ouyang et al., 2011), the levels of serum amino acids in SLE patients were decreased, including some glycolytic and ketogenic amino acids and citric acid, an intermediate metabolite of the tricarboxylic acid cycle, and the decrease in pyruvate, an intermediate product of glycolysis, indicates that SLE patients have energy metabolism disorders. This may be related to the enhanced protein catabolism and increased energy requirements that are present in a state of systemic inflammation.

Notably, cohort-based causal studies between metabolites and SLE are lacking. If differentially abundant metabolites are risk factors or protective factors for SLE, it is meaningful for the prediction of the disease and auxiliary diagnosis based on specific targets. MR analysis uses randomly occurring single nucleotide polymorphisms (SNPs) in human genes as mediation tools. This approach, which is similar to the randomized controlled trial (RCT) design, better ensures the randomization of the sampling. The establishment of exposure factors and outcome variables through instrumental variables can also better prove the causal relationship between them. At the same time, because metabolites may be substances that affect the occurrence of diseases, or may be substances produced after the occurrence of diseases, bidirectional MR analysis can better explain the causal direction of metabolites and diseases.

Therefore, this study collected relatively complete serum metabolome data and introduced MR analysis similar to the RCT design to interpret the causal relationship between SLE and related metabolites through bidirectional MR verification. In this research, MR methodology was used to evaluate the causal effects of genetically proxied metabolomics elements of interest on SLE by selecting metabolite-associated SNPs as instrumental variables (IVs) and to assess pooled metabolic pathways to explain the mechanism.

## 2 Materials and methods

### 2.1 Study design

The dataset that contains all the data in this study is available to the public on the database website. The genome-wide association study (GWAS) summary statistics that have already been published. The ethics committee at each institutional review board authorized all participants’ written informed permission in separate studies. No extra ethical approval or informed consent was required.

**FIGURE 1:**
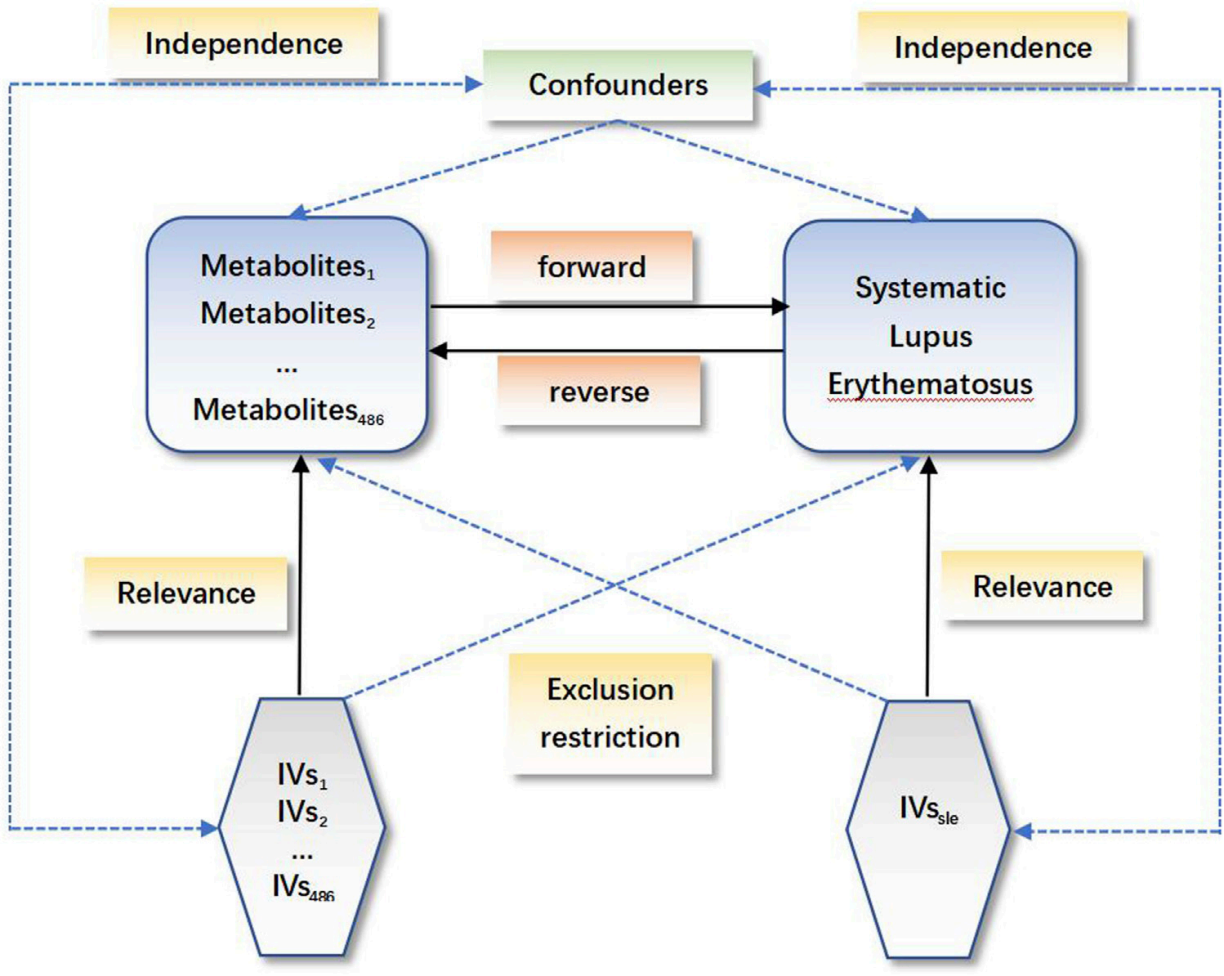
Schematic of the bidirectional Mendelian randomization (MR) analysis. Significant instrumental variables were selected for 486 differentially abundant metabolites and systemic lupus erythematosus, and the bidirectional causalities were probed. The three basic assumptions of MR analysis are illustrated in the acyclic graph.

In the current study, we comprehensively evaluated the relationship between 486 serum metabolites and SLE based on a rigorous MR design. A scientific MR study must include the testing of the following three hypotheses: 1) genetic instrumental variables are strongly associated with the exposure of interest; 2) genetic instrumental variables should be irrelevant to the outcome and independent of any known or unknown confounding factors; and 3) the effect of instrumental variables on the results is mediated only by the interest exposure. Briefly, a bidirectional analysis strategy was utilized to select genetically significant SNPs for 486 human serum metabolites and SLE from a European population. To avoid sample overlap, the metabolites and SLE genetic information selected in this study were obtained from separate GWAS datasets. A schematic of this bidirectional MR study is shown in Figure 1.

### 2.2 GWAS data for human serum metabolites

A genome-wide association aggregate dataset of 486 serum metabolites involved in this study was obtained by Shin et al. (2014). These data are publicly available from the GWAS server (http://metabolomics.helmholtz-muenchen.de/gwas/). The service platform collects relatively complete human serum metabolomics data. A total of 7,824 adults and approximately 2.1 million SNPs from two European cohorts (TwinsUK and KORA cohorts) were included in the GWAS analysis. Of the 486 metabolites, 309 are named metabolites that can be assigned to eight broad metabolic groups (amino acids, carbohydrates, cofactors and vitamins, energy, lipids, nucleotides, peptides, and xenobiotic metabolism), as defined by the kyoto encyclopedia of genes and genomes (KEGG) database (Kanehisa et al., 2012). The chemical properties of another 177 unknown metabolites have not been fully determined.

### 2.3 GWAS data for SLE

The summary data of SLE were obtained from the (https://gwas.mrcieu.ac.uk/) Integrative Epidemiology Unit (IEU) open GWAS project. The GWAS ID is ebi-a-GCST003156 (Bentham et al., 2015). In this GWAS meta-analysis, the summary data included 5,201 SLE cases and 9,066 control cases, yielding a total of 7,071,163 SNPs. We extracted SNPs by analysing vcf format files shared by this platform. The SLE patients from the European population were diagnosed according to the standard American College of Rheumatology classification criteria.

### 2.4 Selection of instrumental variables (IVs)

The selection of IVs in this MR analysis was based on 3 fundamental assumptions. First, for each metabolite, we set *p* < 1 × 10^−5^ as the genome-wide significance threshold to select strongly associated SNPs. Second, a clumping procedure implemented in R software was employed to identify the independent variants. *R* ^2^ < 0.001 within a 500-kilobase (kb) distance was used for linkage-disequilibrium. Finally, to quantitatively verify whether the selected SNPs are strong instruments, we calculated the proportion of phenotypic variation explained (PVE) for each metabolite and the *F* statistic. Typically, a threshold of *F* > 10 is suggested for the following analysis (Burgess et al., 2013).

### 2.5 MR analysis

A standard inverse variance weighted (IVW) method was the main evaluation approach used for causal association exploration between metabolites and SLE (forward MR analysis and reverse MR analysis) in this analysis. MR-Egger and weighted median (WM) were secondary evaluation method. When the instrumental variables satisfy all three major hypotheses, the IVW method can provide a more accurate estimate of the causal effect of exposure and is considered the most efficient MR method. Nevertheless, if some IVs do not conform to the IV hypothesis, the analysis may give inaccurate results. Thus, we implemented the following sensitivity analyses: 1) the *Q* test was carried out with the IVW and MR‒Egger methods to detect possible violations of the hypothesis by the heterogeneity of the correlation between individual IVs (Cohen et al., 2015); 2) the MR‒Egger intercept was implemented to estimate the horizontal pleiotropy, ensuring that the genetic variation was independently related to the metabolite and SLE (Burgess and Thompson, 2017); 3) additional analyses such as the weighted median and weighted mode were applied to enhance the reliability and stability of hypothesis testing; and 4) we conducted an individual SNP analysis and leave-one-out test to evaluate the likelihood of relevance observed by individual SNPs.

### 2.6 Genetic correlation and direction validation

MR analysis may violate cause-effects under the premise of genetic correlation between the exposure and outcome of the research (O'Connor and Price, 2018; Reay et al., 2022). Although SNPs related to SLE were excluded in the selection of IVs, SNPs with no relevance may also impact the occurrence of SLE. Linkage disequilibrium score (LDSC) regression can compute the coinheritance by calling chi-squared statistics based on SNPs (Bulik-Sullivan et al., 2015). Therefore, to ensure that cause-effects were not confused by the coheritability of exposure with the outcome, LDSC was implemented to verify the genetic correlation between the differentially abundant serum metabolites and SLE.

### 2.7 Metabolic pathway analysis

Metabolic pathways were estimated using Web-based metconflict 5.0 (https://www.Metaboanalyst.ca/) (Chong and Xia, 2104). The pathway and enrichment analysis modules were applied to identify probable metabolite clusters or superpathways that may be associated with metabolic processes and the potential association with SLE. The small molecule pathway database (SMPDB) and the KEGG database were applied for reference. The significance level of the pathway was 0.05.

### 2.8 Intersection analysis

An intersection analysis was introduced to analyse the shared metabolites screened by the forward and reverse MR analyses and, in conjunction with potential pathway mechanisms, to evaluate the relationship between metabolic pathways and circulatory deterioration in SLE.

### 2.9 Statistical analysis

All MR analyses were performed using the “TwoSampleMR” package in R (version 4.3.0). LDSC was conducted by the “ldscr” package, and *p* < 0.05 was considered statistically significant. The odds ratio (*OR*) was used to estimate the magnitude and direction of the metabolic impact with its corresponding 95% confidence interval (*CI*).

## 3 Results

### 3.1 Influence of 486 serum metabolites on SLE (forward MR)

As the genome-wide significance threshold was *p* < 1 × 10^−5^ to select strongly associated SNPs, a total of 483 serum metabolites were selected. The IVs contained 7,881 SNPs in total, with a median of 12 SNPs. The F statistic values were all greater than 10, indicating that weak instrumental bias is unlikely to be significant. Except for 176 unnamed metabolites, the remaining 307 metabolites belong to 8 superpathways. The highest proportion of metabolites was lipids (122, 39.74%), followed by amino acids (74, 24.10%) and xenobiotics (39, 12.70%).

**FIGURE 2:**
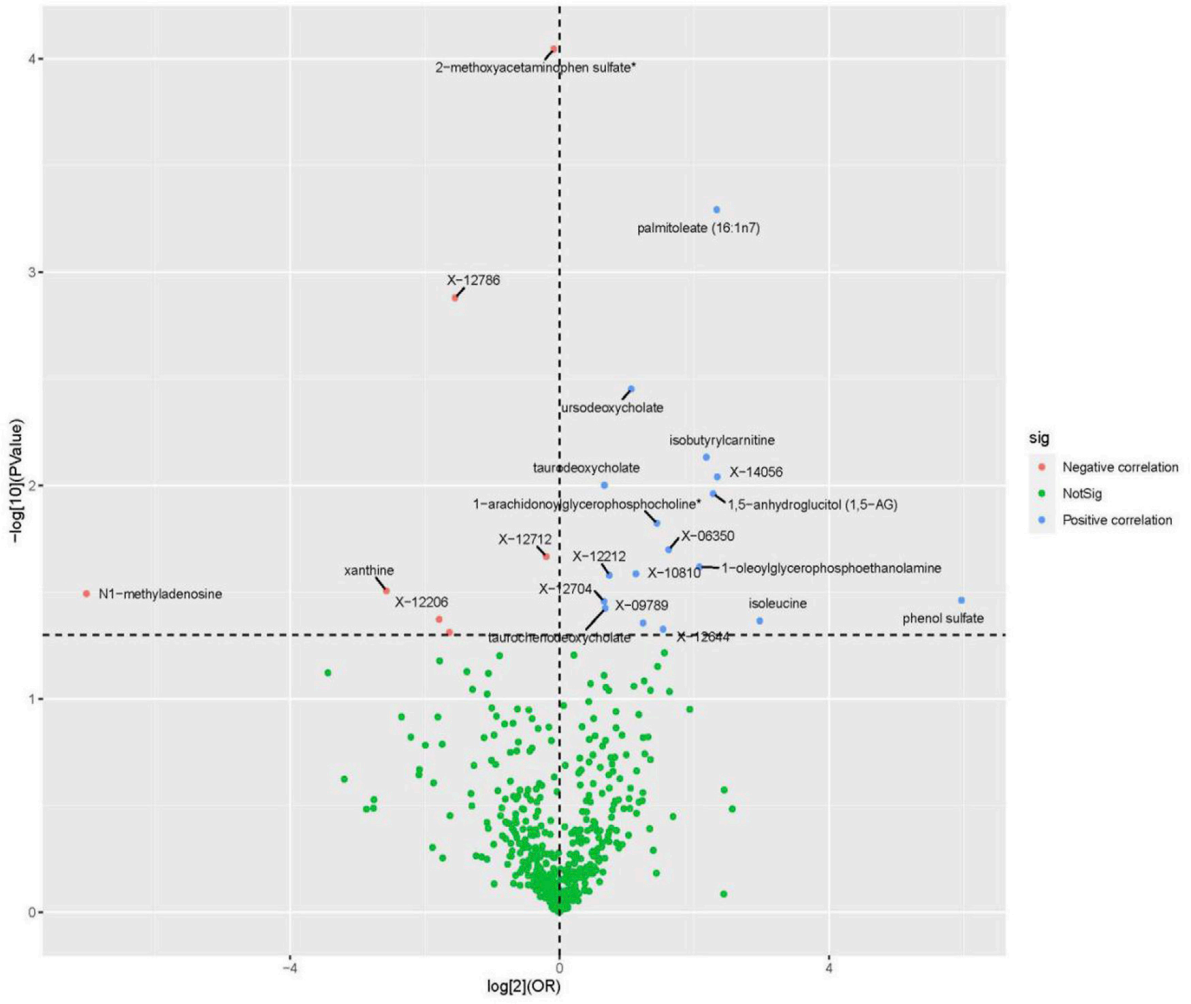
Volcano plot of correlations related to the influence of metabolites on SLE. This plot includes both odds ratios (ORs) in log 2 scale and *p*-values in -log 10 estimated by the inverse variance weighted method for SLE.

**FIGURE 3:**
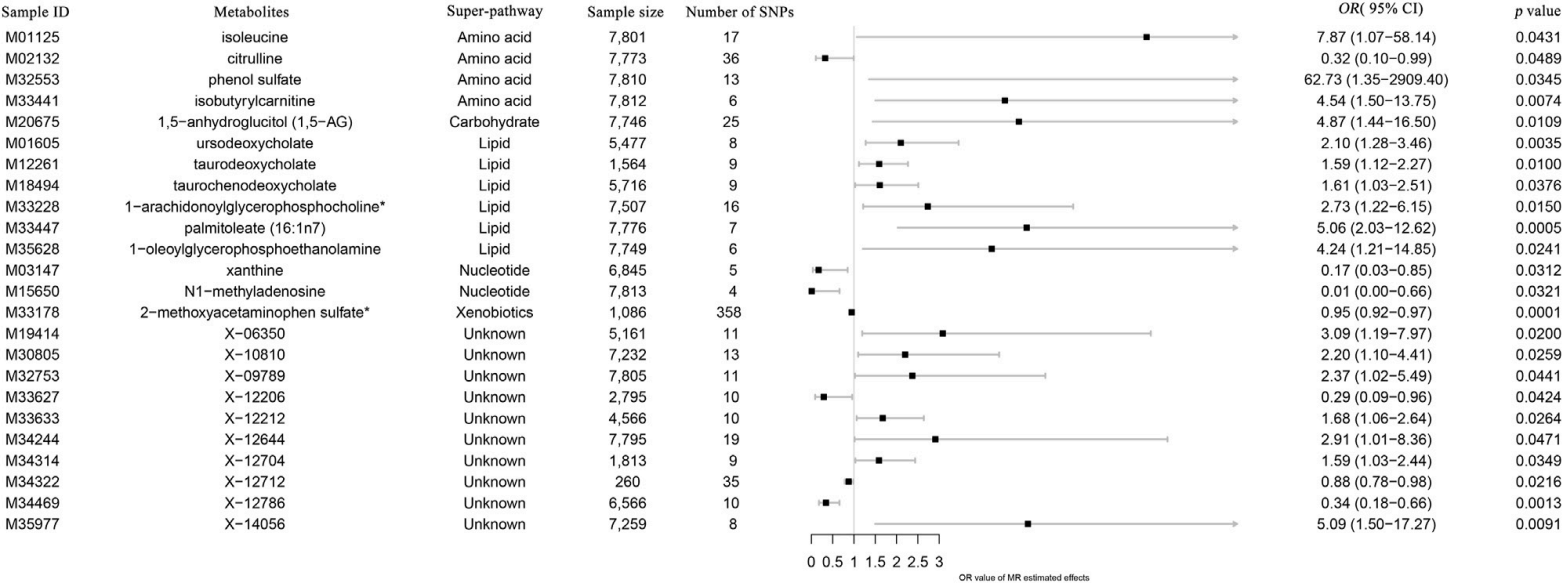
Forest plot of the causal effects of metabolites on the risk of SLE derived from the IVW method. OR, odds ratio; CI, confidence interval.

All metabolic analyses used IVW as the primary analytical methodology, with no evidence of heterogeneity and no weak instruments (Jin et al., 2020). From the primary results, 24 significantly associated metabolites were selected (*p* < 0.05 for IVW), in which 17 were positively associated with SLE and 7 were negatively associated with SLE. 2-Methoxyacetaminophen sulfate (*p* = 8.55 × 10^−5^) was the most significant metabolite, followed by palmitoleate (16:1n7) (*p* = 5.11 × 10^−4^) and ursodeoxycholate (*p* = 3.53 × 10^−3^) (Figure 2). Ten out of the 24 metabolites were unnamed. Among the 14 identified metabolites, 6 metabolites belonging to lipid pathways and 1,5-anhydroglucitol (1,5-AG) belonging to carbohydrate pathways were positively associated with SLE. 2-Methoxyacetaminophen sulfate, belonging to the xenobiotic pathway, exhibits serum protection. In addition to citrulline’s protective effect across the amino acid pathway, its other metabolic involvement may also be a risk factor for SLE (Figure 3). The results of the alternative MR analysis, *Q* test and sensitivity analysis for the 14 known metabolites are shown in Table 1. All IVs passed the sensitivity tests (*p* > 0.05).

**TABLE 1:** The three MR model estimates of the causal relationships between 14 known metabolites and the risk of SLE and tests for heterogeneity and horizontal pleiotropy.

Metabolite	Method	SNP (N)	*OR* (95% *CI*)	*p*	Heterogeneity	*p*	Pleiotropy	*p*
					Q value		Intercept	
Isoleucine	IVW	17	7.87 (1.07–58.14)	0.0431	16.10	0.45	0.03	0.25
	MR–Egger	17	0.60 (0.01–63.39)	0.8311	14.67	0.48		
	WM	17	6.77 (0.34–134.87)	0.2105				
Ursodeoxycholate	IVW	8	2.10 (1.28–3.46)	0.0035	3.39	0.85	0.01	0.70
	MR–Egger	8	1.78 (0.69–4.61)	0.2817	3.23	0.78		
	WM	8	2.00 (1.05–3.80)	0.0340				
Citrulline	IVW	36	0.32 (0.10–0.99)	0.0489	41.70	0.20	−0.02	0.12
	MR–Egger	36	2.29 (0.16–32.33)	0.5427	38.79	0.26		
	WM	36	0.35 (0.07–1.81)	0.2130				
Xanthine	IVW	5	0.17 (0.03–0.85)	0.0312	2.51	0.64	0.01	0.87
	MR–Egger	5	0.12 (0.00–5.06)	0.3510	2.47	0.48		
	WM	5	0.19 (0.02–1.58)	0.1248				
Taurodeoxycholate	IVW	9	1.59 (1.12–2.27)	0.0100	6.46	0.60	−0.02	0.57
	MR–Egger	9	2.10 (0.79–5.60)	0.1798	6.10	0.53		
	WM	9	1.74 (1.11–2.72)	0.0156				
N1-Methyladenosine	IVW	4	0.01 (0.00–0.66)	0.0321	3.34	0.34	0.03	0.66
	MR–Egger	4	0.00 (0.00–1805.66)	0.3965	2.94	0.23		
	WM	4	0.00 (0.00–0.45)	0.0244				
Taurochenodeoxycholate	IVW	9	1.61 (1.03–2.51)	0.0376	6.22	0.62	0.01	0.60
	MR–Egger	9	1.35 (0.62–2.92)	0.4727	5.93	0.55		
	WM	9	2.04 (1.06–3.92)	0.0334				
1,5-Anhydroglucitol (1,5-AG)	IVW	25	4.87 (1.44–16.50)	0.0109	77.30	0.00	0.01	0.83
	MR–Egger	25	3.64 (0.19–70.84)	0.4026	77.14	0.00		
	WM	25	5.12 (1.81–14.51)	0.0021				
Phenol sulfate	IVW	13	62.73 (1.35–2909.40)	0.0345	447.16	0.00	0.14	0.61
	MR–Egger	13	0.95 (0.37–2.43)	0.9207	436.06	0.00		
	WM	13	1.70 (0.00–1916045.26)	0.9419				
2-Methoxyacetaminophen sulfate^a^	IVW	358	0.95 (0.92–0.97)	0.0001	430.43	0.00	0.01	0.48
	MR–Egger	358	0.92 (0.85–1.00)	0.0447	429.81	0.00		
	WM	358	0.94 (0.91–0.98)	0.0047				
1-Arachidonoylglycerophosphocholine^a^	IVW	16	2.73 (1.22–6.15)	0.0150	15.80	0.40	−0.02	0.15
	MR–Egger	16	7.01 (1.65–29.81)	0.0195	13.49	0.49		
	WM	16	3.61 (1.28–10.19)	0.0151				
Isobutyrylcarnitine	IVW	6	4.54 (1.50–13.75)	0.0074	4.25	0.51	0.04	0.44
	MR–Egger	6	1.10 (0.04–34.11)	0.9604	3.52	0.48		
	WM	6	3.36 (0.78–14.49)	0.1044				
Palmitoleate (16:1n7)	IVW	7	5.06 (2.03–12.62)	0.0005	3.07	0.80	−0.03	0.37
	MR–Egger	7	11.64 (1.77–76.43)	0.0508	2.08	0.84		
	WM	7	5.31 (1.53–18.44)	0.0086				
1-Oleoylglycerophosphoethanolamine	IVW	6	4.24 (1.21–14.85)	0.0241	5.08	0.41	−0.11	0.16
	MR–Egger	6	1697.67 (1.68–1715020.92)	0.1028	2.10	0.72		
	WM	6	2.69 (0.47–15.32)	0.2663				

^a^ IVW, inverse variance weighting; WM, weighted median.

The results of LDSC analysis show weak evidence of a genetic correlation between SLE and ursodeoxycholate (*rg* = 0.0395, *se* = 0.2454, *p* = 0.8722), taurodeoxycholate (*rg* = −0.1601, *se* = 0.1715, *p* = 0.3503), N1-methyladenosine (*rg* = −0.0427, *se* = 0.362, *p* = 0.7538), 1,5-anhydroglucitol (1,5-AG) (*rg* = −0.0395, *se* = 0.0840, *p* = 0.6383), 1-arachidonoylglycerophosphocholine (*rg* = −0.0320, *se* = 0.1026, *p* = 0.7554), isobutyrylcarnitine (*rg* = 0.1165, *se* = 0.1219, *p* = 0.3392), palmitoleate (16:1n7) (*rg* = 0.2461, *se* = 0.1551, *p* = 0.1124), and 1-oleoylglycerophosphoethanolamine (*rg* = 0.1058, *se* = 0.2077, *p* = 0.6105), suggesting that the shared genetic component did not confound the MR estimates (Supplementary Table S1).

**FIGURE 4:**
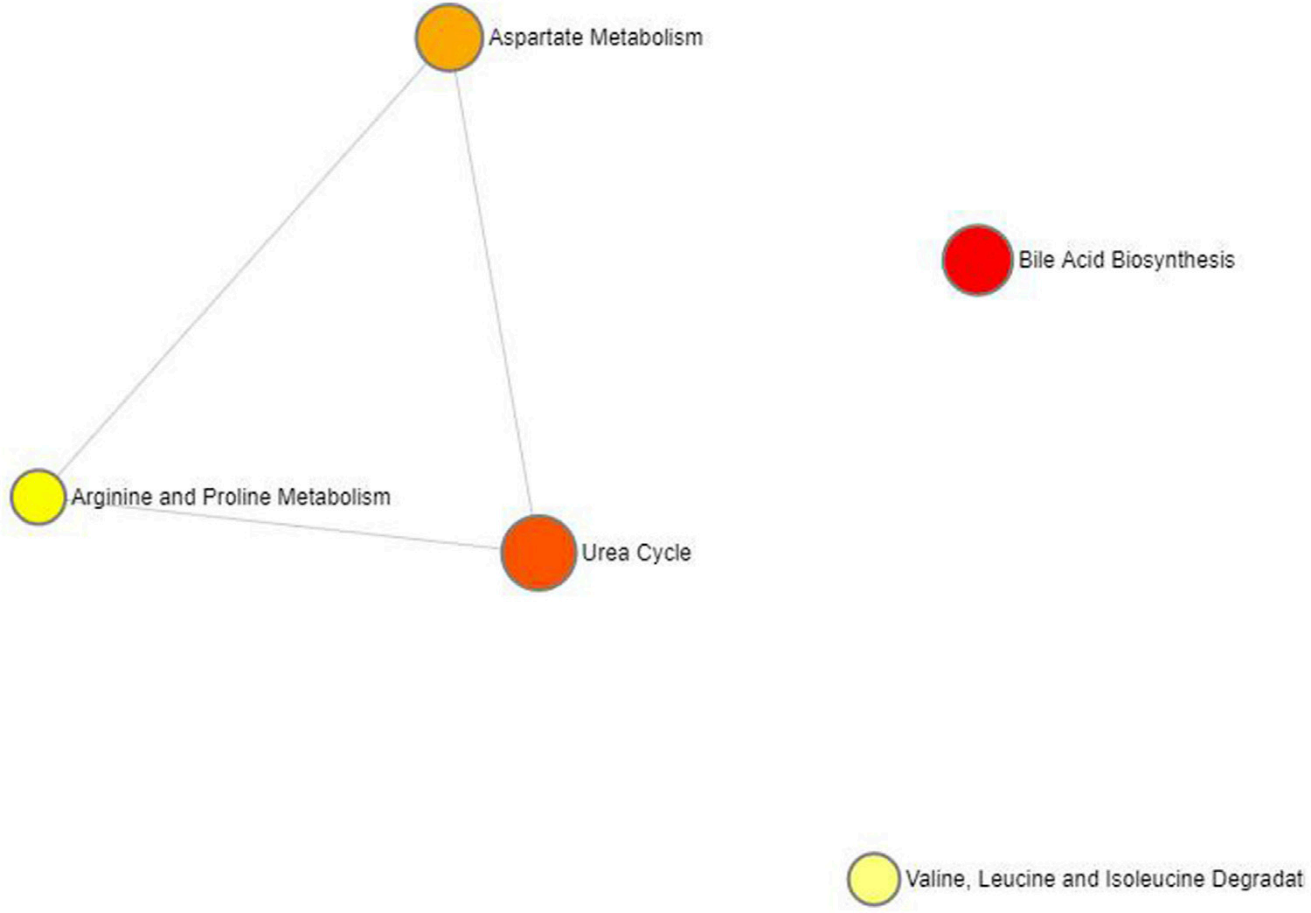
Network of enrichment pathways of the metabolites selected by forward MR. The colour ranges from light yellow to dark red, indicating the level of enrichment significance, and the size of the circle reflects the level of the enrichment ratio.

Fourteen metabolites significantly associated with SLE were entered into the Metabolic Analyzer 5.0 platform to determine various potential metabolic pathways involved in the pathogenesis of SLE. Among them, taurodeoxycholate and taurochenodeoxycholate were involved in the metabolic enrichment pathway of bile acid biosynthesis (*p* = 0.035) (Table 2). The metabolic mechanism formed by the above metabolites may be involved in the pathogenesis of SLE. Figure 4 exhibits the network of interactions among the metabolic pathways involved in this study.

**TABLE 2:** Enrichment pathways of the metabolites selected by forward MR.

Pathway	Total	Expected	Hits	Raw *p*	FDR
Bile acid biosynthesis	65	0.317	2	0.035	1
Urea cycle	29	0.142	1	0.134	1
Aspartate metabolism	35	0.171	1	0.16	1
Arginine and proline metabolism	53	0.259	1	0.234	1
Valine, leucine and isoleucine degradation	60	0.293	1	0.261	1
Purine metabolism	74	0.361	1	0.313	1

### 3.2 Influence of the 486 serum metabolites on SLE (reverse MR)

As the genome-wide significance threshold was *p* < 1 × 10^−5^, 21 significant SNPs were extracted as the IVs for SLE. The 486 serum metabolites were viewed as the outcomes. Of the 486 metabolites, 21 SNPs met the harmonization criteria. In addition, the F statistic values were all greater than 10, indicating that the weak instrumental bias is unlikely to be significant.

**FIGURE 5:**
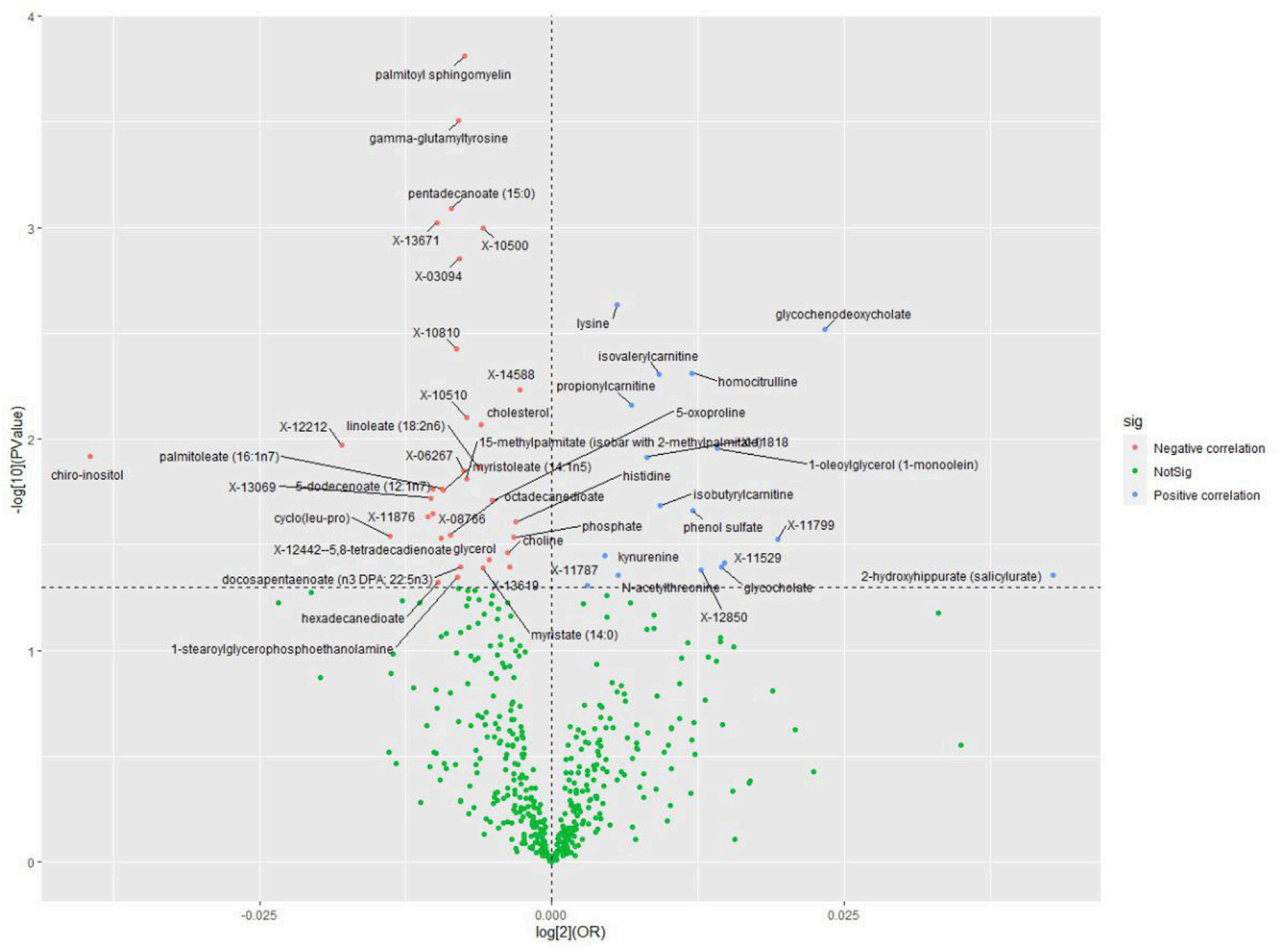
Volcano plot of correlations related to the influence of SLE on metabolites. This plot includes both odds ratios (ORs) in log 2 scale and *p*-values in -log 10 estimated by the inverse variance weighted method for SLE.

**FIGURE 6:**
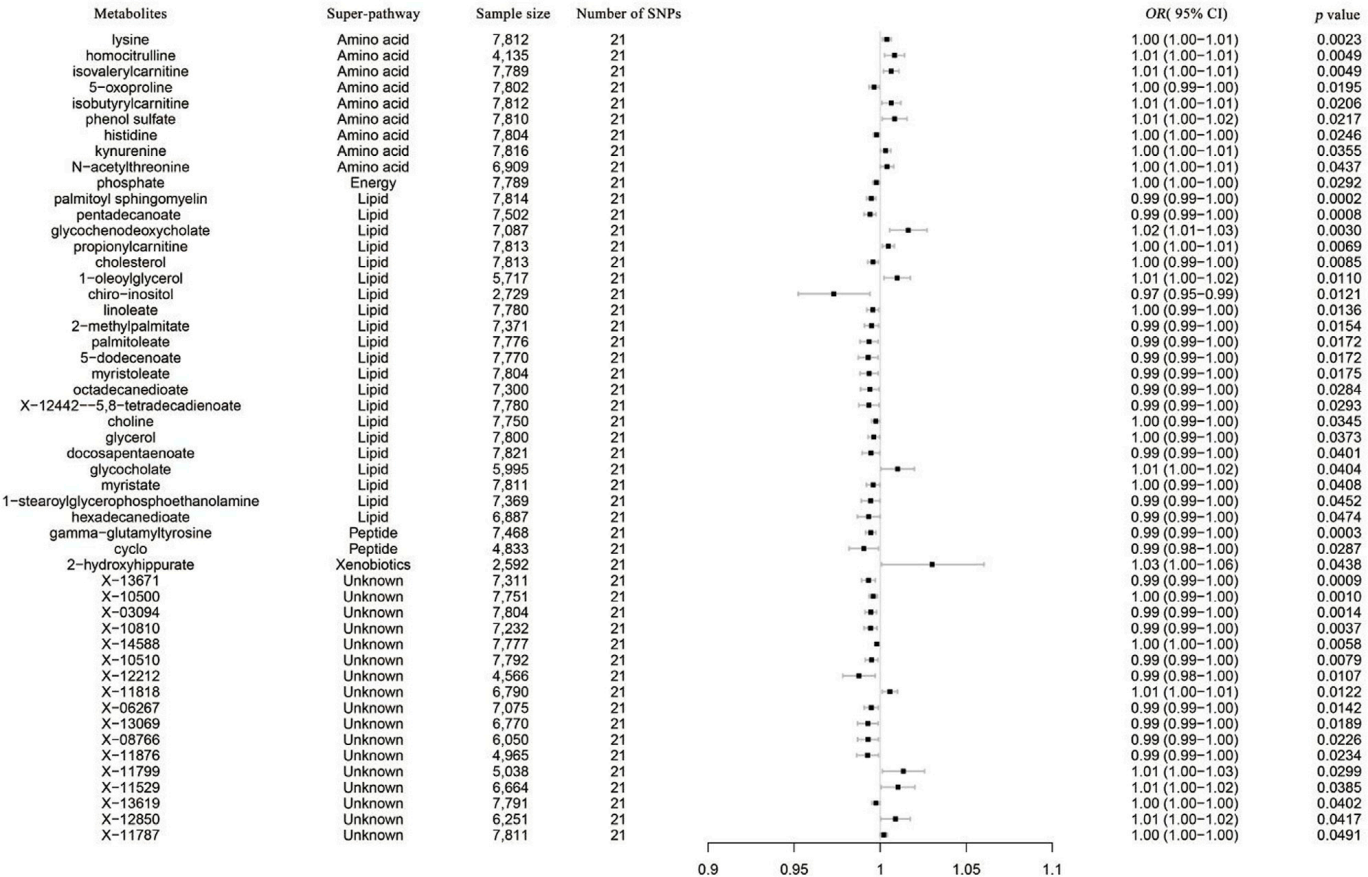
Forest plot of the causal effects of SLE on the risk of metabolites derived from the IVW method. OR, odds ratio; CI, confidence interval.

As no heterogeneity or weak instruments were observed, the IVW method was used as the primary estimation method for SLE casualties. From the primary results, 51 significantly associated metabolites were selected (*p* < 0.05 for IVW), among which 17 were positively associated with SLE, and 34 were negatively associated with SLE. 2-Methoxyacetaminophen sulfate (*p* = 1.55 × 10^−4^) was the most significant metabolite, followed by gamma-glutamyltyrosine (*p* = 3.12 × 10^−4^) and pentadecanoate (15:0) (*p* = 8.12 × 10^−4^) (Figure 5). Seventeen out of the 51 metabolites were unnamed. Among the 34 identified metabolites, 9 metabolites belonging to the amino acid pathway, phosphate belonging to the energy pathway and 2-hydroxyhippurate (salicylurate) belonging to the xenobiotic pathway were positively associated with SLE. Gamma-glutamyltyrosine and cyclo(leu-pro), belonging to the peptide pathway, exhibit serum protection. Twelve out of 21 metabolites across the lipid pathway have a protective effect, and the other 9 metabolites may also be risk factors for SLE (Figure 6). The results of the alternative MR analysis, *Q* test and sensitivity analysis for 14 known metabolites are shown in Table 3. Due to the influence of the number of IVs and the small confidence interval of the *OR* value, the *p*-values of the sensitivity analysis of lysine, 15-methylpalmitate (isobar with 2-methylpalmitate), glycerol and myristate (14:0) are less than 0.05, and a random effect model should be used.

**TABLE 3:** The three MR model estimates of the causal relationships between the risk of SLE and 34 known metabolites and tests for heterogeneity and horizontal pleiotropy.

Metabolite	Method	SNP (N)	*OR* (95% *CI*)	*p*	Heterogeneity	*p*	Pleiotropy	*p*
					Q value (I^2^)		Intercept	
Lysine	IVW	21	1.00 (1.00–1.01)	0.0023	23.01	0.29	0.00	0.00
	MR–Egger	21	1.00 (0.99–1.00)	0.5765	17.48	0.56		
	WM	21	1.00 (1.00–1.01)	0.1123				
Homocitrulline	IVW	21	1.01 (1.00–1.01)	0.0049	18.41	0.56	0.00	0.39
	MR–Egger	21	1.00 (0.99–1.02)	0.5729	17.63	0.55		
	WM	21	1.01 (1.00–1.02)	0.0220				
Isovalerylcarnitine	IVW	21	1.01 (1.00–1.01)	0.0049	18.28	0.57	0.00	0.54
	MR–Egger	21	1.00 (0.99–1.01)	0.4246	17.90	0.53		
	WM	21	1.01 (1.00–1.01)	0.0908				
5-Oxoproline	IVW	21	1.00 (0.99–1.00)	0.0195	32.50	0.04	0.00	0.37
	MR–Egger	21	0.99 (0.99–1.00)	0.0701	31.14	0.04		
	WM	21	1.00 (0.99–1.00)	0.0165				
Isobutyrylcarnitine	IVW	21	1.01 (1.00–1.01)	0.0206	15.47	0.75	0.00	0.12
	MR–Egger	21	1.00 (0.99–1.01)	0.7468	12.81	0.85		
	WM	21	1.00 (1.00–1.01)	0.2638				
Phenol sulfate	IVW	21	1.01 (1.00–1.02)	0.0217	19.39	0.50	0.00	0.11
	MR–Egger	21	1.02 (1.00–1.03)	0.0181	16.54	0.62		
	WM	21	1.01 (1.00–1.02)	0.2566				
Histidine	IVW	21	1.00 (1.00–1.00)	0.0246	12.01	0.92	0.00	0.75
	MR–Egger	21	1.00 (0.99–1.00)	0.1999	11.91	0.89		
	WM	21	1.00 (0.99–1.00)	0.0223				
Kynurenine	IVW	21	1.00 (1.00–1.01)	0.0355	23.51	0.26	0.00	0.39
	MR–Egger	21	1.01 (1.00–1.01)	0.0903	22.59	0.26		
	WM	21	1.00 (1.00–1.01)	0.0426				
N-Acetylthreonine	IVW	21	1.00 (1.00–1.01)	0.0437	25.09	0.20	0.00	0.32
	MR–Egger	21	1.01 (1.00–1.02)	0.0792	23.77	0.21		
	WM	21	1.01 (1.00–1.01)	0.0086				
Phosphate	IVW	21	1.00 (1.00–1.00)	0.0292	15.90	0.72	0.00	0.62
	MR–Egger	21	1.00 (0.99–1.00)	0.1595	15.65	0.68		
	WM	21	1.00 (0.99–1.00)	0.1163				
Palmitoyl sphingomyelin	IVW	21	0.99 (0.99–1.00)	0.0002	20.33	0.44	0.00	0.94
	MR–Egger	21	0.99 (0.99–1.00)	0.0765	20.32	0.38		
	WM	21	0.99 (0.99–1.00)	0.0008				
Pentadecanoate (15:0)	IVW	21	0.99 (0.99–1.00)	0.0008	17.16	0.64	0.00	0.20
	MR–Egger	21	1.00 (0.99–1.01)	0.6735	15.39	0.70		
	WM	21	0.99 (0.99–1.00)	0.0269				
Glycochenodeoxycholate	IVW	21	1.02 (1.01–1.03)	0.0030	15.63	0.74	0.00	0.67
	MR–Egger	21	1.02 (1.00–1.03)	0.0865	15.44	0.69		
	WM	21	1.02 (1.00–1.04)	0.0238				
Propionylcarnitine	IVW	21	1.00 (1.00–1.01)	0.0069	21.21	0.39	0.00	0.74
	MR–Egger	21	1.00 (1.00–1.01)	0.3407	21.08	0.33		
	WM	21	1.00 (1.00–1.01)	0.1212				
Cholesterol	IVW	21	1.00 (0.99–1.00)	0.0085	34.38	0.02	0.00	0.67
	MR–Egger	21	0.99 (0.99–1.00)	0.1279	34.05	0.02		
	WM	21	0.99 (0.99–1.00)	0.0015				
1-Oleoylglycerol (1-monoolein)	IVW	21	1.01 (1.00–1.02)	0.0110	14.46	0.81	0.00	0.36
	MR–Egger	21	1.00 (0.99–1.02)	0.6920	13.59	0.81		
	WM	21	1.01 (1.00–1.02)	0.2091				
Chiro-inositol	IVW	21	0.97 (0.95–0.99)	0.0121	25.39	0.19	0.01	0.51
	MR–Egger	21	0.96 (0.92–1.00)	0.0920	24.82	0.17		
	WM	21	0.95 (0.93–0.98)	0.0010				
Linoleate (18:2n6)	IVW	21	1.00 (0.99–1.00)	0.0136	13.58	0.85	0.00	0.38
	MR–Egger	21	1.00 (0.99–1.01)	0.7048	12.75	0.85		
	WM	21	1.00 (0.99–1.00)	0.1996				
15-Methylpalmitate (isobar with 2-methylpalmitate)	IVW	21	0.99 (0.99–1.00)	0.0154	22.31	0.32	0.00	0.02
	MR–Egger	21	1.00 (1.00–1.01)	0.3099	15.61	0.68		
	WM	21	0.99 (0.99–1.00)	0.0703				
Palmitoleate (16:1n7)	IVW	21	0.99 (0.99–1.00)	0.0172	15.48	0.75	0.00	0.14
	MR–Egger	21	1.00 (0.99–1.01)	0.8296	13.06	0.84		
	WM	21	0.99 (0.99–1.00)	0.0913				
5-Dodecenoate (12:1n7)	IVW	21	0.99 (0.99–1.00)	0.0172	11.49	0.93	0.00	0.10
	MR–Egger	21	1.00 (0.99–1.01)	0.7021	8.51	0.98		
	WM	21	1.00 (0.99–1.00)	0.3315				
Myristoleate (14:1n5)	IVW	21	0.99 (0.99–1.00)	0.0175	17.23	0.64	0.00	0.08
	MR–Egger	21	1.00 (0.99–1.01)	0.6303	13.83	0.79		
	WM	21	1.00 (0.99–1.00)	0.2222				
Octadecanedioate	IVW	21	0.99 (0.99–1.00)	0.0284	16.65	0.68	0.00	0.90
	MR–Egger	21	0.99 (0.98–1.00)	0.2602	16.64	0.61		
	WM	21	0.99 (0.99–1.00)	0.1713				
X-12442-5,8-Tetradecadienoate	IVW	21	0.99 (0.99–1.00)	0.0293	13.37	0.86	0.00	0.83
	MR–Egger	21	0.99 (0.98–1.01)	0.4072	13.33	0.82		
	WM	21	1.00 (0.99–1.00)	0.2668				
Choline	IVW	21	1.00 (0.99–1.00)	0.0345	29.26	0.08	0.00	0.86
	MR–Egger	21	1.00 (0.99–1.00)	0.4353	29.22	0.06		
	WM	21	1.00 (1.00–1.00)	0.3815				
Glycerol	IVW	21	1.00 (0.99–1.00)	0.0373	17.46	0.62	0.00	0.04
	MR–Egger	21	1.00 (1.00–1.01)	0.3491	12.58	0.86		
	WM	21	1.00 (0.99–1.00)	0.4697				
Docosapentaenoate (n3 DPA; 22:5n3)	IVW	21	0.99 (0.99–1.00)	0.0401	15.58	0.74	0.00	0.71
	MR–Egger	21	0.99 (0.99–1.00)	0.5293	15.44	0.69		
	WM	21	1.00 (0.99–1.01)	0.1263				
Glycocholate	IVW	21	1.01 (1.00–1.02)	0.0404	25.23	0.19	0.00	0.35
	MR–Egger	21	1.00 (0.98–1.02)	0.8721	24.07	0.19		
	WM	21	1.01 (1.00–1.02)	0.1093				
Myristate (14:0)	IVW	21	1.00 (0.99–1.00)	0.0408	23.64	0.26	0.00	0.02
	MR–Egger	21	1.00 (1.00–1.01)	0.2770	17.40	0.56		
	WM	21	1.00 (0.99–1.00)	0.2438				
1-Stearoylglycerophosphoethanolamine	IVW	21	0.99 (0.99–1.00)	0.0452	23.01	0.29	0.00	0.95
	MR–Egger	21	0.99 (0.98–1.01)	0.3953	23.00	0.24		
	WM	21	0.99 (0.99–1.00)	0.0405				
Hexadecanedioate	IVW	21	0.99 (0.99–1.00)	0.0474	15.95	0.72	0.00	0.36
	MR–Egger	21	1.00 (0.99–1.01)	0.9022	15.08	0.72		
	WM	21	0.99 (0.98–1.00)	0.0496				
Gamma-glutamyltyrosine	IVW	21	0.99 (0.99–1.00)	0.0003	20.69	0.42	0.00	0.19
	MR–Egger	21	0.99 (0.98–1.00)	0.0088	18.86	0.47		
	WM	21	0.99 (0.99–1.00)	0.0104				
Cyclo (leu-pro)	IVW	21	0.99 (0.98–1.00)	0.0287	17.13	0.64	0.00	0.97
	MR–Egger	21	0.99 (0.97–1.01)	0.2919	17.13	0.58		
	WM	21	0.99 (0.98–1.00)	0.0747				
2-Hydroxyhippurate (salicylurate)	IVW	21	1.03 (1.00–1.06)	0.0438	20.31	0.44	0.00	0.92
	MR–Egger	21	1.03 (0.97–1.10)	0.3152	20.30	0.38		
	WM	21	1.02 (0.98–1.06)	0.3609				

^a^ IVW, inverse variance weighting; WM, weighted median.

The results of the LDSC analysis show weak evidence of a genetic correlation between 36 metabolites and SLE (15 metabolites could not be used to estimate heritability because the genetic covariance matrix includes traits estimated to have negative heritability). *Rg* ranges from −0.3351 to 1.0997, the standard error from 0.0710 to 0.5204, and the *p*-value from 0.0023 to 0.9950. Except for phosphate belonging to the energy pathway (*p* = 0.0024) and X-13671 (unknown) (*p* = 0.0064), the genetic correlation of the other metabolites was not significant (*p* > 0.05), suggesting that these shared genetic components did not confound the MR estimates (Supplementary Table S2).

**FIGURE 7:**
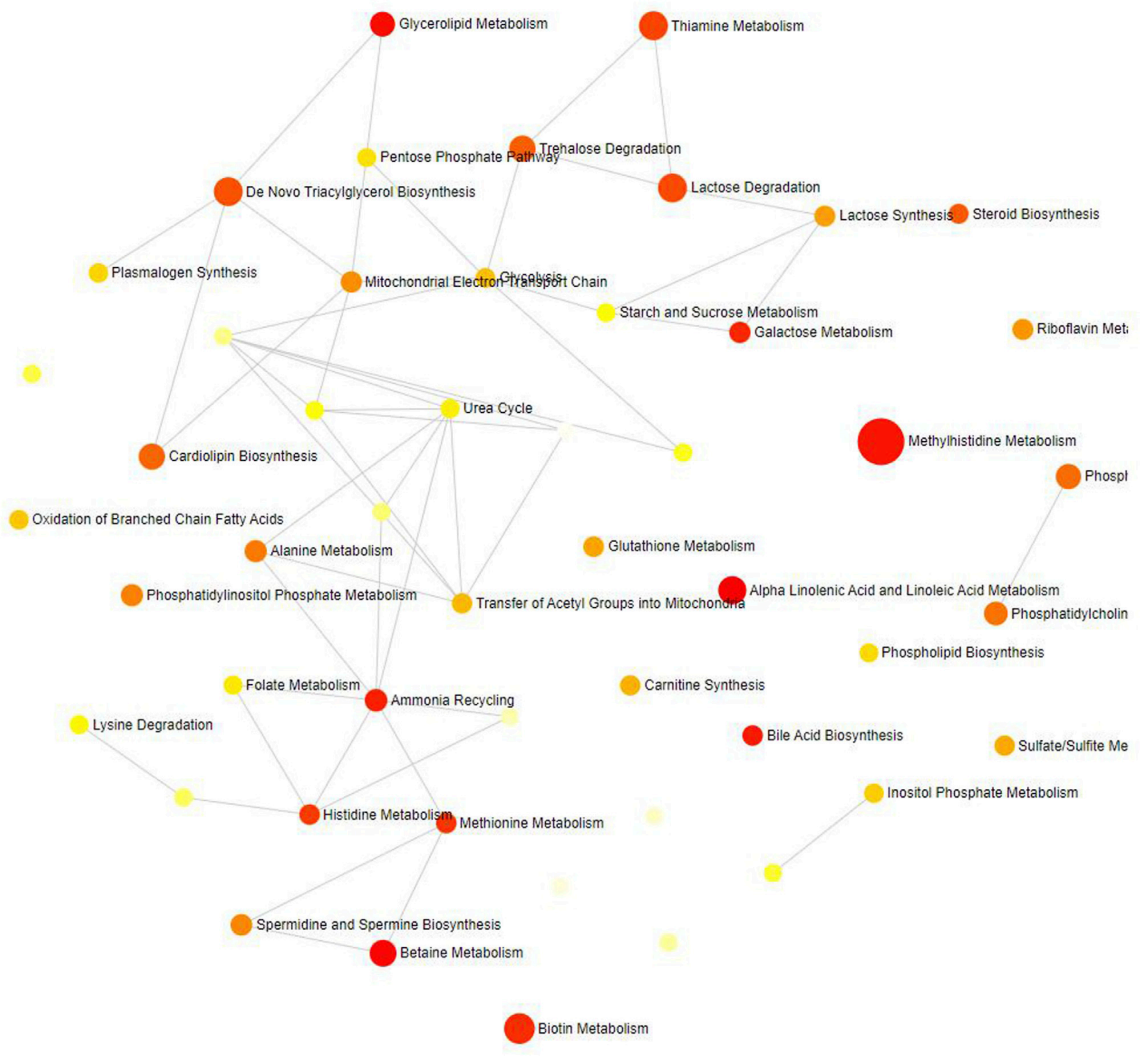
Network of enrichment pathways of the metabolites selected by reverse MR. The colour ranges from light yellow to dark red, indicating the level of enrichment significance, and the size of the circle reflects the level of the enrichment ratio.

Thirty-three metabolites significantly influenced by SLE were put into the Metabolic Analyzer 5.0 platform to determine various potential metabolic pathways involved in the pathogenesis of SLE and immunomics. Among them, linoleic acid and docosapentaenoic acid (22n-6) were involved in the metabolic pathway of alpha-linolenic acid and linoleic acid metabolism (*p =* 0.0260), choline and phosphate were involved in the metabolic pathway of betaine metabolism (*p =* 0.0314), and glycerol and phosphate were involved in the metabolic pathway of glycerolipid metabolism (*p =* 0.0435) (Table 4). The metabolic mechanism formed by the above metabolites may be involved in the pathogenesis impacted by SLE. Figure 7 exhibits the network of interactions among the metabolic pathways involved in this analysis.

**TABLE 4:** Top ten enrichment pathways of the metabolites selected by the reverse MR.

Pathway	Total	Expected	Hits	Raw *p*	FDR
Alpha-linolenic acid and linoleic acid metabolism	19	0.26	2	0.026	0.825
Betaine metabolism	21	0.287	2	0.0314	0.825
Glycerolipid metabolism	25	0.342	2	0.0435	0.825
Methylhistidine metabolism	4	0.0547	1	0.0537	0.825
Bile acid biosynthesis	65	0.889	3	0.0537	0.825
Ammonia recycling	32	0.438	2	0.0681	0.825
Galactose metabolism	38	0.52	2	0.0922	0.825
Biotin metabolism	8	0.109	1	0.105	0.825
Methionine metabolism	43	0.588	2	0.114	0.825

### 3.3 Intersection between forward MR and reverse MR

**FIGURE 8:**
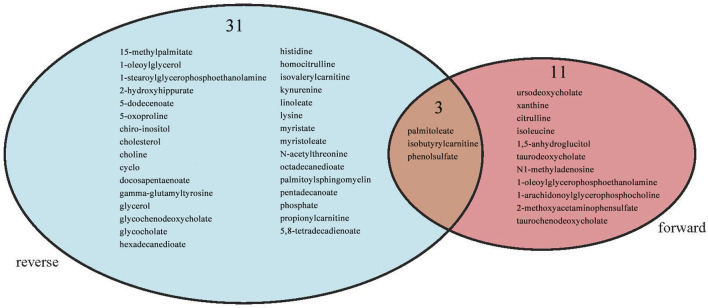
Intersection between forward MR and reverse MR.

Intersection analysis was introduced to analyse the shared metabolites screened by the forward and reverse MR analyses. Palmitoleate belonging to the lipid superpathway and isobutyrylcarnitine and phenol sulfate belonging to the amino acid superpathway appeared in the intersection (Figure 8).

## 4 Discussion

We elucidated the bidirectional causal relationship between metabolites and SLE by using genetic variation as the IVs in a two-sample MR model. We discovered that 24 metabolites belonging to the lipid, carbohydrate, xenobiotic and amino acid superpathways may increase the risk of SLE occurrence. In addition, the metabolic disorders of 51 metabolites belonging to the amino acid, energy, xenobiotics, peptide and lipid superpathways were affected by SLE. Palmitoleate belonging to the lipid superpathway and isobutyrylcarnitine and phenol sulfate belonging to the amino acid superpathway were factors with two-way causation.

In the first part of this study (forward MR), the metabolism of bile acids has aroused our great concern. Our study showed that the significantly altered metabolites (taurodeoxycholate, taurochenodeoxycholate, and ursodeoxycholate) were mapped to bile acid metabolism pathways (*p* = 0.035).

There is a subtle relationship between bile acid metabolism, gut microbes and the immune system (Cai et al., 2022; Collins et al., 2023). Bile acids are involved in processes including glucose and lipid metabolism, energy balance, and immune regulation through several key receptors such as the farnesoid X receptor (FXR), Takeda G protein-coupled receptor 5 (TGR5), pregnane X receptor (PXR), vitamin D receptor (VDR), sphingosine-1-phosphate receptor 2(SP1PR2), and retinoid-related orphan receptorγt (RORγt) (Jia et al., 2023). In the T cells of the intestine, bile acids affect the differentiation of Th17, Treg through the regulation of RORγt, FXR, and VDR receptors (Hang et al., 2019). Through Spearman’s correlation analysis showed good prediction of the SLEDAI score by bile acids (deoxycholic acid, glycocholic acid, and isohyodeoxycholic acid) and arachidonic acid, demonstrating a correlation between bile acids and SLE activity in a metabolomics study (He et al., 2020a). Our MR analysis indicated that individuals with high levels of bile acids (ursodeoxycholate, taurodeoxycholate, taurochenodeoxycholate) were more likely to develop SLE. Previous research on SLE found that chenodeoxycholic acid (CDCA) suppresses inflammatory cytokines and showing its association with SLE in MRL/lpr mice (Lian et al., 2012). Considering that bile acids are involved in the metabolic balance and immunoregulatory processes in multiple tissues, the exploration of the mechanism affecting systemic lupus erythematosus through the regulation of bile acids and their receptors will be the next step of our research.

Altered lipid metabolism is well known to occur in SLE patients. Especially in serum, a metabolomics study revealed that the identified lipids accounted for approximately 46.3% of the total identified metabolites, and the altered lipids in SLE patients were more than 65% of all changed metabolites (de Carvalho et al., 2008). Two observational studies from China and Brazil suggested that SLE can cause disorders of lipid metabolism [12,28]. In the present systematic MR study, we confirmed these associations of lipid metabolism (palmitoleat, isobutyrylcarnitine, 1-arachidonoylglycerophosphocholine, 1-oleoylglycerophosphoethanolamine) with the risk of SLE from a genetic perspective.

Citrullline was found to be protective as a differential metabolite in our MR analyses (*p* = 0.0489). No direct literature reports of citrulline and SLE have been retrieved. But previous studies have shown that increased citrulline directly binds to JAK2 and inhibits JAK2-STAT1 signalling (Nakayamada and Tanaka, 2022). The JAK-STAT signalling pathway is involved in the pathogenesis of multiple autoimmune diseases, including SLE. In an animal model of lupus, the JAK inhibitor tofacitinib improved clinical features, immune deregulation, and vascular dysfunction. It can therefore be speculated that increased citrulline, as a JAK2 inhibitor, may act as a protective factor in the pathogenesis of SLE.

1,5-AG is one of the main polyol sugars in the human body, is mainly derived from food and is hardly synthesized by the human body (Yamanouchi and Akanuma, 1994). When blood glucose levels are above the renal glucose threshold, the reabsorption of 1,5-AG in the kidney is competitively inhibited by glucose, which leads to a decrease in serum 1,5-AG levels. Measuring serum 1,5-AG levels can reflect the average blood glucose level in the last 1–2 weeks (Almeida et al., 2021). Previous studies showed that elevated levels of 1,5-AG in the immune microenvironment could inhibit macrophage proinflammatory polarization and promote survival of acute B lymphocyte leukemia cells *in vitro* through upregulation of C-X-C Motif Chemokine Ligand 14 (CXCL14) (Wu et al., 2023b). Our MR study identified that 1,5-AG acted as a pathogenic factor, leading to a high risk of SLE (*p* = 0.0109). Considering that the detection technology of 1, 5-AG is relatively stable and mature (Ortiz-Martínez et al., 2022), it has the potential to be used as a biomarker for predicting SLE to achieve clinical translation.

In a metabolomics study covering 4,569 differentially expressed metabolites identified in SLE, elevated levels of these metabolites were associated with the biosynthesis of valine, leucine, and isoleucine (Zeng et al., 2021). This study indicated that the metabolism of isoleucine is involved in the pathogenesis of SLE. By comparison, our MR study identified that isoleucine (*p* = 0.0431) also acted as a pathogenic factor, leading to a high risk of SLE.

For the other metabolites we screened, such as 2-methoxyacetaminophen sulfate, xanthine, N1-methyladenosine, we had not found reports related to SLE. A mounting array of studies has revealed that patients with SLE have abnormal metabolism compared to the general population (Teng et al., 2020). Due to limited understanding of the role about these metabolite in SLE, it would be a suggestive finding.

In the second part of this analysis (reverse MR), we found that alpha-linolenic acid and linoleic acid were the most significantly enriched metabolic pathways (*p* = 0.0260). The linolenic acid involved in this pathway is a new class of oxylipins that are produced by enzymatic or nonenzymatic oxidation of polyunsaturated fatty acids (PUFAs) (Kortz et al., 2014). Oxidized lipids play a very important role in the pathological and physiological environment, especially in inflammation and the immune response (Fullerton et al., 2014; Dennis and Norris, 2015). In SLE, several oxylipins have been identified to be significantly changed and critical for SLE pathophysiology, suggesting the potential utility of oxylipins as candidate biomarkers for SLE diagnosis and clinical treatment (Hye Khan et al., 2019; He et al., 2020b). Jingquan H et al. used targeted mass spectrometry analysis to explore the alteration of oxylipins in the serum of 98 SLE patients and 106 healthy controls (He et al., 2022). The negative correlation of linolenic acid with lupus nephritis and SLE disease activity is consistent with our study. This study further confirmed that SLE may be the cause of reduced linolenic acid metabolism.

This reverse MR analysis also revealed enrichment of the betaine metabolism pathway that was significantly associated with SLE (*p* = 0.0314). Although we did not find reports of a direct association between betaine and SLE, a study based on ultra-high-performance liquid chromatography-quadrupole time-of-flight mass spectrometry suggested that the betaine pathway differed significantly between rheumatoid arthritis patients and healthy controls (Zhu et al., 2022). Rheumatoid arthritis and SLE are autoimmune-related diseases. In fact, these two diseases share several clinical manifestations, serological profiles, and immunological characteristics. The third significant metabolic enrichment pathway of interest in the reverse MR analysis was glycerolipid metabolism (*p* = 0.0435). Glycerolipids are an important component of the cell membrane. Their activation not only has antioxidant effects (Matsufuji et al., 2000) but also has anti-inflammatory effects, especially in atherosclerosis caused by inflammatory factors (KaranionisH et al., 2002). It is well known that atherosclerosis is a major complication of SLE (Liu et al., 2022). The levels of circulating apoptotic endothelial cells are increased in SLE patients and are strongly linked to vascular dysfunction and increased tissue factor levels, suggesting an imbalance between endothelial cell damage and repair in individuals with SLE (Rajagopalan et al., 2004). The regulation of glycerolipids, as intermediate metabolites, is undoubtedly an innovative idea to prevent atherosclerosis in SLE patients.

In addition to the above significantly enriched pathways, this study specifically focused on three metabolites (palmitoleate, isobutyrylcarnitine and phenol sulfate) with bidirectional causal effects on SLE. Palmitoleic acid is a kind of free fatty acids (FFAs), and its correlation with SLE has been studied. A study used gas chromatography with mass spectrometry to identify the relationship between 24 FFAs and SLE (Shin et al., 2017). The results showed that palmitoleic acid levels were significantly elevated in SLE patients. Our causal study suggests that the elevation of palmitoleic acid levels may be due to metabolic abnormalities caused by the SLE immune response. In a study of serum metabolites and acute graft-versus-host disease (GVHD), isobutyrylcarnitine was found to be altered in patients with advanced GVHD (Reikvam et al., 2016). Carnitine is important for fatty acid transport and may also be important for the release of immunomodulatory cytokines. There is a lack of direct reports on the relationship between phenol sulfate and SLE. However, phenol sulfate is the main metabolite of intestinal microorganisms (Lustgarten et al., 2014), and the balance of intestinal microorganisms is closely related to the pathogenicity of SLE (Manfredo Vieira et al., 2018). Under the influence of the gut microbiota, the microbial pathways related to sulfur metabolism are altered in SLE patients (Tomofuji et al., 2021). In short, the above three metabolites may be involved in the whole process of SLE onset and development, and their metabolic mechanisms interact with SLE-related mechanisms.

Our study has several merits. In this observational study, the application of MR methodology efficiently decreases the potential confounding factors and reverses causality. The chronology of the causal chain of metabolites and SLE is clearer. Second, because a large sample for the MR analysis was recruited in this study, the statistical power of the results was increased. Third, to avoid bias from population stratification, this research limited the GWAS data to the major European ancestry cohorts. Furthermore, we adopted a two-way MR analysis process to more comprehensively explain the causal relationship between metabolites and SLE, as well as the mechanisms of the metabolic abnormalities associated with SLE onset and disease progression.

There are certain limitations to this study. First, the study was carried out using summary datasets, which makes it challenging to undertake stratified analysis. Some behavioural and biological characteristics are obscured. Second, it is possible that some metabolites associated with SLE may be excluded due to the level of pleiotropy of the IVs. Third, some important metabolites and pathways are ignored because they are not named or annotated in the pathway database. Therefore, the unproven metabolites still need to be explored in depth. In the future, we will continue to pay attention to the database, especially the characteristics of different types of SLE, and make more accurate prediction of influencing factors.

In conclusion, the results of this study indicate that the levels of some particular metabolites may either contribute to the immune response inducing SLE or may be intermediates in the development and progression of SLE. These metabolites can be used as auxiliary diagnostic tools for SLE and for the evaluation of disease progression and therapeutic effects. Future studies are necessary to derive more accurate inferences from our findings.

## Data Availability

The datasets presented in this study can be found in online repositories. The names of the repository/repositories and accession number(s) can be found in the article/Supplementary Material.

## Abbreviations

SLE, Systemic Lupus Erythematosus; RCT, Randomized Controlled Trial; MR, Mendelian Randomization; IVs, Instrumental Variables; GWAS, Genome-Wide Association Study; SNPs, Single Nucleotide Polymorphisms; LDSC, Linkage Disequilibrium Score; IVW, Inverse Variance Weighting; WM, Weighted Median; KEGG, Kyoto Encyclopedia of Genes and Genomes; SMPDB, Small Molecule Pathway Database; GAPDH, glyceraldehyde-3-phosphate dehydrogenase; IFN-γ, Interferon γ; FXR, farnesoid X receptor; TGR5, Takeda G protein-coupled receptor 5; PXR, pregnane X receptor; VDR, vitamin D receptor; SP1PR2, sphingosine-1-phosphate receptor 2; RORγt, retinoid-related orphan receptorγt; SLEDAI, systemic lupus erythematosus disease activity index; CXCL14, C-X-C Motif Chemokine Ligand 14; 1,5-AG, 1,5-anhydroglucitol; PUFAs, Polyunsaturated Fatty Acids; CDCA, chenodeoxycholic acid; FFAs, Free Fatty Acids; GVHD, Graft-versus-host Disease.
